# Crystal structure and Hirshfeld surface analysis of a pyridiniminium bromide salt: 1-[2-(adamantan-1-yl)-2-oxoeth­yl]pyridin-4-iminium bromide

**DOI:** 10.1107/S2056989018009131

**Published:** 2018-06-28

**Authors:** Huey Chong Kwong, Imdad Mahmud Pathi, C. S. Chidan Kumar, Ching Kheng Quah, Md. Azharul Arafath

**Affiliations:** a School of Chemical Sciences, Universiti Sains Malaysia, 11800 USM, Penang; b X-ray Crystallography Unit, School of Physics, Universiti Sains Malaysia, 11800 USM, Penang, Malaysia; c Department of Engineering Chemistry, Vidya Vikas Institute of Engineering & Technology, Visvesvaraya Technological University, Alanahalli, Mysuru 570028, Karnataka, India; d Department of Chemistry, Shahjalal University of Science and Technology, Sylhet 3114, Bangladesh

**Keywords:** crystal structure, pyridiniminium salt, hydrogen bonding, Hirshfeld surface analysis

## Abstract

In the cation of the title salt, C_17_H_23_N_2_O^+^·Br^−^, the adamantyl moiety and the pyridiniminium ring are inclined to the ketone bridge by torsion angles of −78.1 (2) (C—C—C=O) and 58.3 (2)° (C—C—N—C), respectively, and the ketone bridge has a C—C—C—N torsion angle of 174.80 (15)°. In the crystal, the cations are connected into chains parallel to the *c* axis by C—H⋯O hydrogen bonds. The chains are further linked into layers parallel to the *bc* plane by N—H⋯Br and C—H⋯Br hydrogen bonds, C—H⋯π inter­actions and π–π stacking inter­actions [centroid-to-centroid distance = 3.5657 (11) Å]. A Hirshfeld surface analysis, which comprises the *d*_norm_ surface, electrostatic potential map and two-dimensional fingerprint plots, was carried out to verify the contribution of the various inter­molecular inter­actions.

## Abstract 2

The asymmetric unit of the title pyridiniminium halide salt comprise of one cation and one anion. In the crystal, mol­ecules are linked by N—H⋯Br and C—H⋯O hydrogen bonds, C—H⋯π inter­actions, and π–π inter­actions into layers. The inter­molecular inter­actions in the crystal structure are qu­anti­fied by Hirshfeld surface analysis.

## Chemical context   

Adamantane derivatives have been shown to exhibit various biological activities such as anti­viral (Zoidis *et al.*, 2010 ▸), anti-diabetic (Zettl *et al.*, 2010 ▸), anti­microbial (Piérard *et al.*, 2009 ▸), anti-inflammatory (Lamanna *et al.*, 2012 ▸), anti­oxidant (Priyanka *et al.*, 2013 ▸) and central nervous system activities (Reisberg *et al.*, 2003 ▸). Besides, adamantane-based chemotherapeutics have been developed for treating viral infections, for example influenza A, herpes simplex and HIV (Liu *et al.*, 2011 ▸). There are a number of negatively charged enzymes and cofactors and many diseases, including cystic fibrosis, have been found to result from defects in the ion channel function (Ashcroft, 1999 ▸). The anion–π non-covalent inter­action has been explored both theoretically and experimentally and selective anion receptors and channels have been designed (Ballester, 2008 ▸; Schottel *et al.*, 2008 ▸; Hay & Bryantsev, 2008 ▸; Frontera *et al.*, 2011 ▸).

Ionic liquids (ILs) have attracted a lot of inter­est over the past decade because of their unusual range of properties such as negligible vapour pressure, excellent thermal stability in a wide temperature range, no flammability and high ionic conductivity (Davis, 2004 ▸). ILs are excellent alternatives to volatile organic compounds (VOCs). An ionic liquid has a strong solvation ability and can dissolve polar and non-polar species with efficient selectivity, which can be modified by changing the anion (Blanchard *et al.*, 2001 ▸). ILs have been used successfully as solvents in several reactions such as isomerization, dimerization, hydrogenation, and Heck and Suzuki coupling reactions (Chauvin & Olivier-Bourbigou, 1995 ▸; Holbrey & Seddon, 1999 ▸). They have also performed well as solvents in bio-catalysed and homogeneous catalytic reactions, and can be used as lubricants to wet the surface of metals, polymers and inorganic materials (Crosthwaite *et al.*, 2004 ▸).
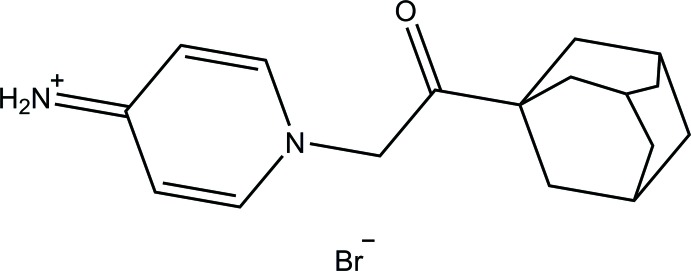


## Structural commentary   

**Figure 1:**
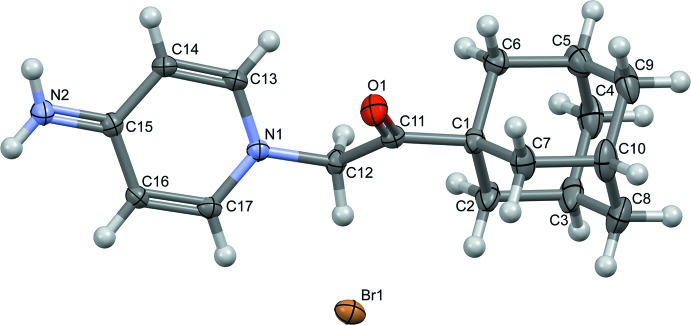
The mol­ecular structure of the title salt with displacement ellipsoids drawn at the 50% probability level.

Fig. 1 ▸ shows the asymmetric unt of the title salt, which consists of a 1-[2-(adamantan-1-yl)-2-oxoeth­yl]pyridin-4-iminium cation and a bromide anion. The cation is constructed from an adamantyl moiety (C1–C10) and a pyridiniminium ring (N1/C13–C17), which are connected by a ketone bridge [(C11=O1)—C12]. The bond angles formed by the quaternary carbon (C1) with the surrounding secondary carbons (C2, C6 and C7) are comparable with those reported for related structures which range from 107.40 (12) to 110.82 (13)° (Rouchal *et al.*, 2011 ▸)*.* Both the adamantyl and pyridiniminium rings are twisted away from the ketone bridge to reduce repulsion, as indicated by the torsion angles C6—C1—C11=O1 [−78.1 (2)°] and C11—C12—N1—C13 [58.3 (2)°]. The ketone bridge is in an *anti­periplanar* conformation [C1—C11—C12—N1 = 174.80 (15)°]. The dihedral angle formed by the pyrimidinium ring with the ketone bridge is 59.77 (14)°. Bond lengths and angles in the cation are within normal ranges (Allen, 2002 ▸). However, the N2—C15 bond length [1.325 (2) Å] is shorter than expected for an NH_2_—C_ar_ single bond [1.38 (3) Å], indicating partial double-bond character. Similar bond lengths are found in related compounds with an N^+^=C double bond (Chidan Kumar *et al.*, 2017 ▸; Sharmila *et al.*, 2014 ▸; Yue *et al.*, 2013 ▸).

## Supra­molecular features   

**Figure 2:**
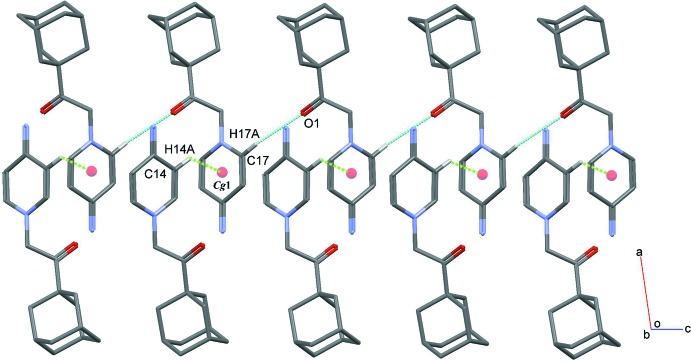
Partial packing diagram of the the cations showing the C17—H17*A*⋯O1 hydrogen bonds (blue dashed lines) and the C14—H14*A*⋯π inter­actions (green dashed lines).

In the crystal, the cations are linked into chains along the *c-*axis direction *via* C17—H17*A*⋯O1 hydrogen bonds (Table 1 ▸, Fig. 2 ▸). The chains inter­act through N—H⋯Br and C—H⋯Br hydrogen bonds to form layers parallel to the *bc* plane, which are further enforced by C—H⋯π and π–π inter­actions [centroid-to-centroid distance 3.5657 (11) Å].

## Hirshfeld Surface Analysis   

**Figure 3:**
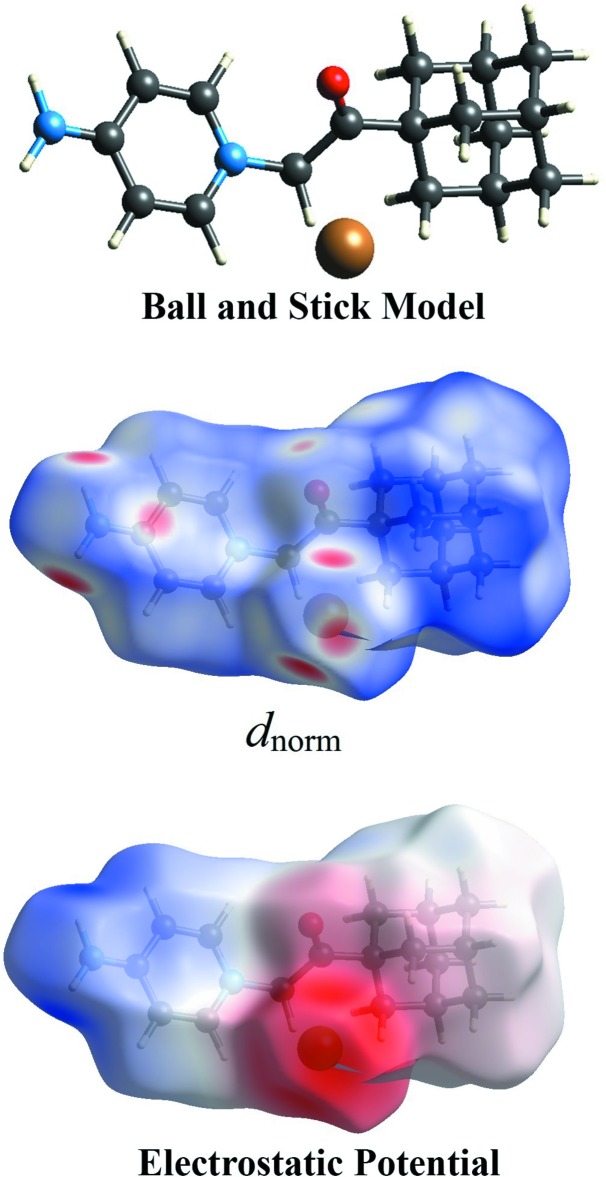
Hirshfeld surfaces mapped over *d*_norm_ and electrostatic potential to visualize the inter­molecular contacts in the title salt. The mol­ecule in the ball-and-stick model is in the same orientation shown in the Hirshfeld surface and electrostatic potential plots.

The Hirshfeld surface analysis (Spackman & Jayatilaka, 2009 ▸) of the title salt was performed using *CrystalExplorer3.1* (Wolff *et al.*, 2012 ▸), and comprises *d*_norm_ surface plots, electrostatic potentials and two-dimensional fingerprint plots (Spackman & McKinnon, 2002 ▸). The ball-and-stick model, *d*_norm_ surface and electrostatic potential plots of the title salt are shown in Fig. 3 ▸. Those plots were generated in order to qu­antify and give visual confirmation of the inter­molecular inter­actions and to explain the observed crystal packing. The dark-red spots on the *d*_norm_ surface arise because of short inter­atomic contacts, while the other weak inter­molecular inter­actions appear as light-red spots. Furthermore, the negative electrostatic potential (red region) in the electrostatic potential map indicates hydrogen-acceptor potential, whereas the hydrogen donors are represented by positive electrostatic potential (blue region) (Spackman *et al.*, 2008 ▸).

**Figure 4:**
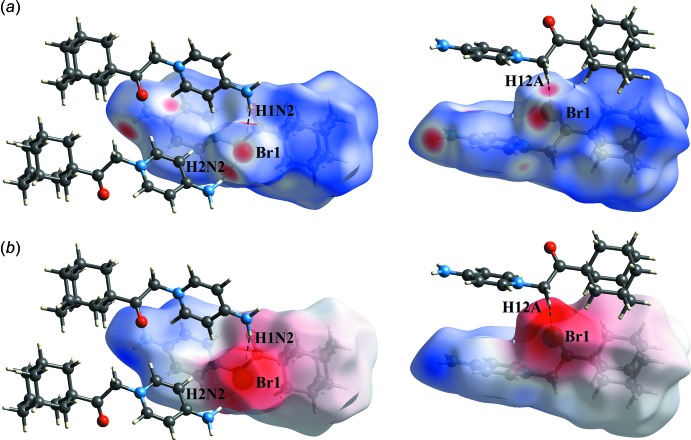
A visualization of the N—H⋯Br and C—H⋯Br inter­actions. (*a*) *d*_norm_ and (*b*) electrostatic potential mapped on Hirshfeld surfaces in order to visualize the N—H⋯Br and C—H⋯Br inter­actions (black dotted lines).

**Figure 5:**
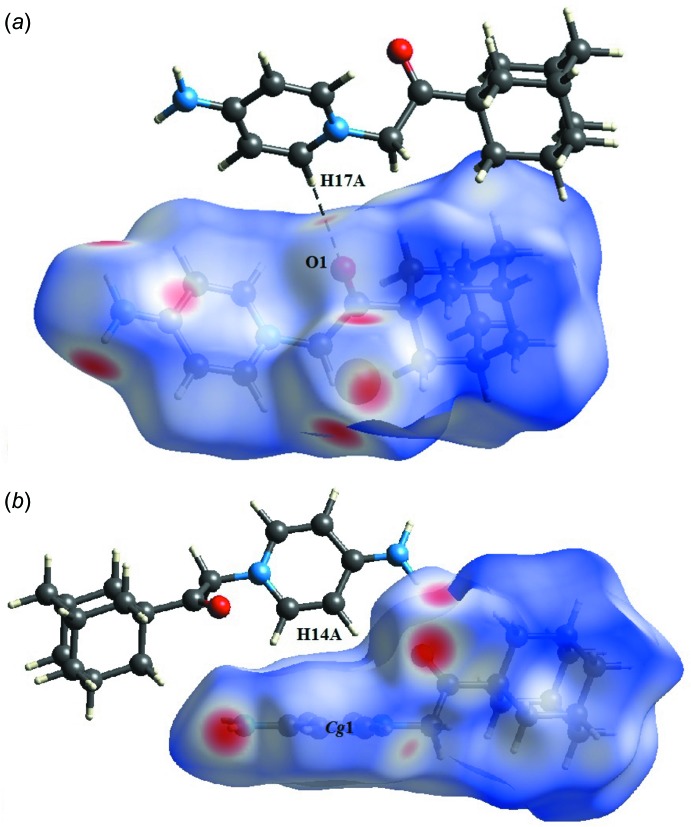
*d*_norm_ mapped on Hirshfeld surfaces in order to visualize (*a*) the C—H⋯Br hydrogen bond (black dashed line) and (*b*) the C—H⋯π inter­actions.

Dark-red spots that are close to atoms H1*N*2, H2*N*2, H12*A* and Br1 in the *d_norm_* surface mapping are the result of the N2—H1*N*2⋯Br1, N2—H2*N*2⋯Br1 and C12—H12*A*⋯Br1 hydrogen bonds (Fig. 4 ▸ *a*). This observation is further confirmed by the respective electrostatic potential maps where Br1 shows negative electrostatic potential as a hydrogen acceptor (red region, Fig. 4 ▸ *b*). Beside those two short inter­molecular contacts, the C—H⋯O and C—H⋯π inter­actions are shown as light-red spots on the *d*_norm_ surface (Fig. 5 ▸).

**Figure 6:**
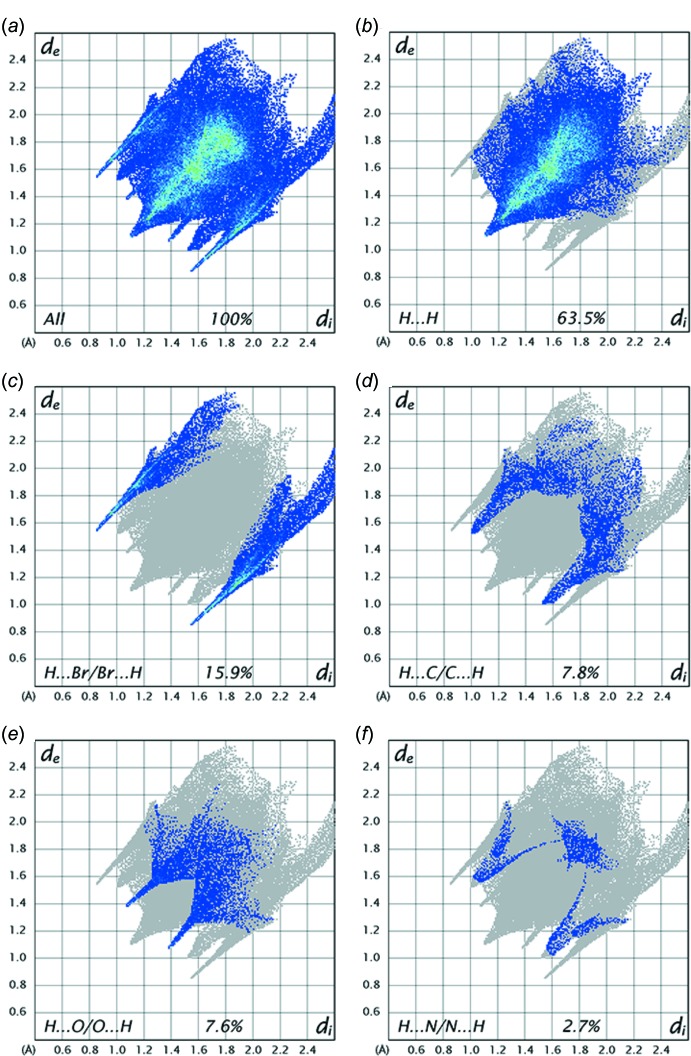
Fingerprint plots.

A qu­anti­tative analysis of the inter­molecular inter­actions can be made by studying the fingerprint plots (FP); characteristic pseudo-symmetry wings in the *d*_e_ and *d*_i_ diagonal axes can be seen in the overall two-dimensional FP (Fig. 6 ▸). The most significant inter­molecular inter­actions are the H⋯H inter­actions (63.5%), which appear in the central region of the FP with *d*_e_ = *d*_i_ ≃ 2.2 Å (Fig. 6 ▸ *b*). The reciprocal H⋯Br/Br⋯H and H⋯O/O⋯H inter­actions with 15.9% and 7.6% contributions, respectively are present as sharp symmetrical spikes at *d*_e_ + *d*_i_ ≃ 2.4 and 2.5 Å, respectively (Fig. 6 ▸ *c* and 6*e*). The reciprocal H⋯C/C⋯H inter­actions appear as two symmetrical narrow wings at *d*_e_ + *d*_i_ ≃ 2.5 Å and contribute 7.8% to the Hirshfeld surface (Fig. 6 ▸ *d*). The reciprocal N⋯H/H⋯N interactions appear as a symmetrical V-shaped wing in the FP map with *d*_e_ + *d*_i_ ≃ 2.7 Å and contribute 2.7% to the Hirshfeld surface (Fig. 6 ▸ *f*). The percentage contributions for other inter­molecular contacts are less than 2.6%.

## Synthesis and crystallization   

A mixture of 1-adamantly bromo­methyl ketone (2.75 g, 10 mmol) and 4-amino­pyridine (0.11 g, 1 mmol) was dissolved in 10 ml of toluene at room temperature, followed by stirring at 358 K for 18 h. The completion of the reaction was marked by the amount of the separated solid from the initially clear and homogeneous mixture of the starting materials. The solid was filtered and washed by ethyl acetate. The final pyridiniminium salt was obtained after the solid had been dried under reduced pressure to remove all volatile organic compounds (Said *et al.*, 2017 ▸; Sheshadri *et al.*, 2018 ▸). Plate-like colourless crystals were obtained by slow evaporation of an acetone solution.

## Refinement   

Crystal data, data collection and structure refinement details are summarized in Table 2 ▸. C-bound H atoms were positioned geometrically [C—H = 0.93–0.98 Å] and refined using a riding model with *U*_iso_(H) = 1.2*U*_eq_(C). The N-bound H atoms were located in a difference-Fourier map and freely refined. One outlier (100) was omitted in the last cycles of refinement.

## Supplementary Material

Crystal structure: contains datablock(s) I. DOI: 10.1107/S2056989018009131/rz5239sup1.cif

Structure factors: contains datablock(s) I. DOI: 10.1107/S2056989018009131/rz5239Isup2.hkl

Supporting information file. DOI: 10.1107/S2056989018009131/rz5239Isup3.cml

CCDC reference: 1851334

Additional supporting information:  crystallographic information; 3D view; checkCIF report

## Figures and Tables

**Table 1:** Hydrogen-bond geometry (Å, °) *Cg*1 is the centroid of the N1/C13–C17 ring.

*D*—H⋯*A*	*D*—H	H⋯*A*	*D*⋯*A*	*D*—H⋯*A*
N2—H1*N*2⋯Br1^i^	0.84 (2)	2.73 (2)	3.499 (2)	153 (2)
N2—H2*N*2⋯Br1^ii^	0.85 (2)	2.56 (2)	3.393 (2)	169 (2)
C12—H12*A*⋯Br1^iii^	0.97	2.72	3.664 (2)	166
C17—H17*A*⋯O1^iv^	0.93	2.59	3.434 (2)	150
C14—H14*A*⋯*Cg*1^i^	0.93	2.94	3.608 (2)	130

Symmetry codes: (i) 
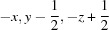
; (ii) 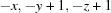; (iii) 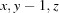; (iv) 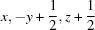.

**Table 2:** Experimental details

Crystal data	
Chemical formula	C_17_H_23_N_2_O^+^·Br^−^
*M*_r_	351.28
Crystal system, space group	Monoclinic, *P*2_1_/*c*
Temperature (K)	294
*a*, *b*, *c* (Å)	18.758 (2), 7.1508 (8), 11.9909 (14)
β (°)	98.2117 (17)
*V* (Å^3^)	1591.9 (3)
*Z*	4
Radiation type	Mo *K*α
μ (mm^−1^)	2.58
Crystal size (mm)	0.38 × 0.25 × 0.09

Data collection	
Diffractometer	Bruker APEXII DUO CCD area-detector
Absorption correction	Multi-scan (*SADABS*; Bruker, 2012 ▸)
*T*_min_, *T*_max_	0.320, 0.408
No. of measured, independent and observed [*I* > 2σ(*I*)] reflections	35418, 4897, 3392
*R*_int_	0.051
(sin θ/λ)_max_ (Å^−1^)	0.716

Refinement	
*R*[*F* ^2^ > 2σ(*F* ^2^)], *wR*(*F* ^2^), *S*	0.035, 0.080, 1.01
No. of reflections	4897
No. of parameters	198
H-atom treatment	H atoms treated by a mixture of independent and constrained refinement
Δρ_max_, Δρ_min_ (e Å^−3^)	0.39, −0.25

Computer programs: *APEX2* and *SAINT* (Bruker, 2012 ▸), *SHELXS97* (Sheldrick, 2008 ▸), *SHELXL2013* (Sheldrick, 2015 ▸), *Mercury* (Macrae *et al.*, 2006 ▸) and *PLATON* (Spek, 2009 ▸).

## supplementary crystallographic information

### Crystal data

**Table:**

C_17_H_23_N_2_O^+^·Br^−^	*F*(000) = 728
*M**_r_* = 351.28	*D*_x_ = 1.466 Mg m^−^^3^
Monoclinic, *P*2_1_/*c*	Mo *K*α radiation, λ = 0.71073 Å
*a* = 18.758 (2) Å	Cell parameters from 7278 reflections
*b* = 7.1508 (8) Å	θ = 3.1–25.2°
*c* = 11.9909 (14) Å	µ = 2.58 mm^−^^1^
β = 98.2117 (17)°	*T* = 294 K
*V* = 1591.9 (3) Å^3^	Plate, colourless
*Z* = 4	0.38 × 0.25 × 0.09 mm

### Data collection

**Table:**

Bruker APEXII DUO CCD area-detector diffractometer	4897 independent reflections
Radiation source: fine-focus sealed tube	3392 reflections with *I* > 2σ(*I*)
Graphite monochromator	*R*_int_ = 0.051
φ and ω scans	θ_max_ = 30.6°, θ_min_ = 2.2°
Absorption correction: multi-scan (SADABS; Bruker, 2012)	*h* = −26→26
*T*_min_ = 0.320, *T*_max_ = 0.408	*k* = −10→10
35418 measured reflections	*l* = −17→17

### Refinement

**Table:**

Refinement on *F*^2^	0 restraints
Least-squares matrix: full	Hydrogen site location: mixed
*R*[*F*^2^ > 2σ(*F*^2^)] = 0.035	H atoms treated by a mixture of independent and constrained refinement
*wR*(*F*^2^) = 0.080	*w* = 1/[σ^2^(*F*_o_^2^) + (0.0364*P*)^2^ + 0.3328*P*] where *P* = (*F*_o_^2^ + 2*F*_c_^2^)/3
*S* = 1.01	(Δ/σ)_max_ = 0.001
4897 reflections	Δρ_max_ = 0.39 e Å^−^^3^
198 parameters	Δρ_min_ = −0.25 e Å^−^^3^

### Special details

**Table:**

Experimental. The following wavelength and cell were deduced by SADABS from the direction cosines etc. They are given here for emergency use only: CELL 0.71078 12.009 7.162 18.799 89.983 98.202 90.025
Geometry. All esds (except the esd in the dihedral angle between two l.s. planes) are estimated using the full covariance matrix. The cell esds are taken into account individually in the estimation of esds in distances, angles and torsion angles; correlations between esds in cell parameters are only used when they are defined by crystal symmetry. An approximate (isotropic) treatment of cell esds is used for estimating esds involving l.s. planes.

### Fractional atomic coordinates and isotropic or equivalent isotropic displacement parameters (Å^2^)

**Table:**

	*x*	*y*	*z*	*U*_iso_*/*U*_eq_	
Br1	0.18668 (2)	0.58832 (3)	0.43377 (2)	0.04161 (8)	
O1	0.16460 (7)	0.2172 (2)	0.22373 (11)	0.0449 (3)	
N1	0.09073 (8)	0.11925 (19)	0.39566 (12)	0.0287 (3)	
N2	−0.12926 (9)	0.1691 (3)	0.35275 (16)	0.0399 (4)	
H1N2	−0.1522 (14)	0.122 (3)	0.294 (2)	0.060 (8)*	
H2N2	−0.1491 (11)	0.232 (3)	0.3994 (18)	0.046 (6)*	
C1	0.28202 (10)	0.1231 (3)	0.31057 (14)	0.0334 (4)	
C2	0.32236 (11)	0.1050 (4)	0.43067 (16)	0.0487 (6)	
H2A	0.3014	0.0049	0.4699	0.058*	
H2B	0.3180	0.2204	0.4717	0.058*	
C3	0.40229 (11)	0.0632 (4)	0.4260 (2)	0.0617 (7)	
H3A	0.4278	0.0525	0.5029	0.074*	
C4	0.40873 (13)	−0.1190 (4)	0.3647 (2)	0.0647 (7)	
H4A	0.3876	−0.2193	0.4034	0.078*	
H4B	0.4591	−0.1483	0.3636	0.078*	
C5	0.37011 (12)	−0.1023 (4)	0.2448 (2)	0.0551 (6)	
H5A	0.3751	−0.2198	0.2045	0.066*	
C6	0.28984 (11)	−0.0621 (3)	0.24763 (17)	0.0433 (5)	
H6A	0.2687	−0.1634	0.2855	0.052*	
H6B	0.2646	−0.0533	0.1713	0.052*	
C7	0.31564 (11)	0.2816 (3)	0.2499 (2)	0.0513 (5)	
H7A	0.3111	0.3987	0.2891	0.062*	
H7B	0.2906	0.2941	0.1737	0.062*	
C8	0.43485 (13)	0.2227 (5)	0.3659 (3)	0.0765 (8)	
H8A	0.4306	0.3390	0.4060	0.092*	
H8B	0.4856	0.1988	0.3639	0.092*	
C9	0.40260 (13)	0.0572 (4)	0.1841 (2)	0.0672 (8)	
H9A	0.4531	0.0318	0.1808	0.081*	
H9B	0.3779	0.0676	0.1076	0.081*	
C10	0.39530 (12)	0.2385 (4)	0.2467 (2)	0.0653 (7)	
H10A	0.4166	0.3403	0.2077	0.078*	
C11	0.20180 (10)	0.1573 (3)	0.30656 (14)	0.0317 (4)	
C12	0.16924 (9)	0.0976 (3)	0.40968 (15)	0.0301 (4)	
H12A	0.1813	−0.0324	0.4260	0.036*	
H12B	0.1905	0.1715	0.4738	0.036*	
C13	0.04945 (10)	0.0291 (3)	0.31040 (15)	0.0357 (4)	
H13A	0.0718	−0.0446	0.2616	0.043*	
C14	−0.02299 (10)	0.0429 (3)	0.29395 (15)	0.0353 (4)	
H14A	−0.0498	−0.0198	0.2341	0.042*	
C15	−0.05818 (10)	0.1526 (3)	0.36771 (14)	0.0295 (4)	
C16	−0.01401 (9)	0.2433 (2)	0.45605 (14)	0.0300 (4)	
H16A	−0.0348	0.3164	0.5069	0.036*	
C17	0.05879 (10)	0.2251 (2)	0.46772 (14)	0.0297 (4)	
H17A	0.0872	0.2867	0.5264	0.036*	

### Atomic displacement parameters (Å^2^)

**Table:**

	*U*^11^	*U*^22^	*U*^33^	*U*^12^	*U*^13^	*U*^23^
Br1	0.05492 (14)	0.03289 (11)	0.03750 (11)	0.00147 (9)	0.00823 (8)	−0.00127 (8)
O1	0.0371 (8)	0.0609 (10)	0.0352 (7)	0.0074 (7)	0.0001 (6)	0.0094 (7)
N1	0.0278 (8)	0.0256 (8)	0.0330 (7)	−0.0007 (6)	0.0051 (6)	−0.0020 (6)
N2	0.0300 (9)	0.0507 (11)	0.0389 (9)	0.0022 (8)	0.0051 (8)	−0.0078 (8)
C1	0.0276 (9)	0.0426 (11)	0.0295 (8)	−0.0005 (8)	0.0028 (7)	0.0024 (7)
C2	0.0304 (10)	0.0816 (17)	0.0330 (9)	−0.0033 (11)	0.0009 (8)	−0.0015 (10)
C3	0.0273 (11)	0.109 (2)	0.0454 (12)	0.0014 (13)	−0.0049 (9)	0.0035 (13)
C4	0.0344 (12)	0.092 (2)	0.0681 (16)	0.0187 (13)	0.0077 (11)	0.0252 (14)
C5	0.0452 (13)	0.0659 (16)	0.0550 (13)	0.0171 (11)	0.0094 (10)	−0.0039 (11)
C6	0.0355 (11)	0.0506 (13)	0.0427 (10)	0.0056 (9)	0.0018 (8)	−0.0041 (9)
C7	0.0434 (12)	0.0528 (14)	0.0613 (13)	0.0005 (10)	0.0198 (10)	0.0138 (11)
C8	0.0323 (13)	0.102 (2)	0.096 (2)	−0.0162 (14)	0.0110 (13)	−0.0112 (18)
C9	0.0450 (14)	0.105 (2)	0.0562 (14)	0.0200 (14)	0.0234 (11)	0.0144 (14)
C10	0.0453 (14)	0.0729 (18)	0.0839 (18)	−0.0032 (12)	0.0307 (13)	0.0217 (15)
C11	0.0312 (10)	0.0329 (9)	0.0309 (9)	−0.0004 (8)	0.0035 (7)	−0.0012 (7)
C12	0.0248 (9)	0.0298 (9)	0.0357 (8)	0.0041 (7)	0.0042 (7)	0.0028 (7)
C13	0.0352 (10)	0.0347 (10)	0.0380 (9)	−0.0021 (8)	0.0076 (8)	−0.0130 (8)
C14	0.0345 (10)	0.0373 (10)	0.0342 (9)	−0.0045 (8)	0.0052 (8)	−0.0107 (8)
C15	0.0307 (10)	0.0277 (8)	0.0308 (8)	0.0011 (7)	0.0067 (7)	0.0031 (7)
C16	0.0336 (10)	0.0288 (9)	0.0290 (8)	0.0024 (8)	0.0089 (7)	−0.0017 (7)
C17	0.0362 (10)	0.0256 (8)	0.0275 (8)	−0.0022 (7)	0.0053 (7)	−0.0013 (7)

### Geometric parameters (Å, º)

**Table:**

O1—C11	1.208 (2)	C6—H6A	0.9700
N1—C17	1.352 (2)	C6—H6B	0.9700
N1—C13	1.354 (2)	C7—C10	1.531 (3)
N1—C12	1.466 (2)	C7—H7A	0.9700
N2—C15	1.325 (2)	C7—H7B	0.9700
N2—H1N2	0.85 (3)	C8—C10	1.517 (4)
N2—H2N2	0.84 (2)	C8—H8A	0.9700
C1—C11	1.518 (3)	C8—H8B	0.9700
C1—C7	1.530 (3)	C9—C10	1.514 (4)
C1—C2	1.534 (3)	C9—H9A	0.9700
C1—C6	1.542 (3)	C9—H9B	0.9700
C2—C3	1.538 (3)	C10—H10A	0.9800
C2—H2A	0.9700	C11—C12	1.517 (2)
C2—H2B	0.9700	C12—H12A	0.9700
C3—C4	1.509 (4)	C12—H12B	0.9700
C3—C8	1.523 (4)	C13—C14	1.348 (3)
C3—H3A	0.9800	C13—H13A	0.9300
C4—C5	1.520 (3)	C14—C15	1.414 (2)
C4—H4A	0.9700	C14—H14A	0.9300
C4—H4B	0.9700	C15—C16	1.407 (2)
C5—C9	1.526 (4)	C16—C17	1.359 (2)
C5—C6	1.538 (3)	C16—H16A	0.9300
C5—H5A	0.9800	C17—H17A	0.9300
			
C17—N1—C13	119.43 (15)	C1—C7—H7B	109.8
C17—N1—C12	121.03 (15)	C10—C7—H7B	109.8
C13—N1—C12	119.53 (15)	H7A—C7—H7B	108.2
C15—N2—H1N2	117.3 (18)	C10—C8—C3	109.1 (2)
C15—N2—H2N2	119.2 (14)	C10—C8—H8A	109.9
H1N2—N2—H2N2	123 (2)	C3—C8—H8A	109.9
C11—C1—C7	109.85 (16)	C10—C8—H8B	109.9
C11—C1—C2	113.40 (15)	C3—C8—H8B	109.9
C7—C1—C2	109.10 (17)	H8A—C8—H8B	108.3
C11—C1—C6	106.61 (15)	C10—C9—C5	109.47 (19)
C7—C1—C6	109.20 (16)	C10—C9—H9A	109.8
C2—C1—C6	108.58 (17)	C5—C9—H9A	109.8
C1—C2—C3	109.56 (16)	C10—C9—H9B	109.8
C1—C2—H2A	109.8	C5—C9—H9B	109.8
C3—C2—H2A	109.8	H9A—C9—H9B	108.2
C1—C2—H2B	109.8	C9—C10—C8	109.7 (2)
C3—C2—H2B	109.8	C9—C10—C7	110.0 (2)
H2A—C2—H2B	108.2	C8—C10—C7	109.8 (2)
C4—C3—C8	110.5 (2)	C9—C10—H10A	109.1
C4—C3—C2	109.4 (2)	C8—C10—H10A	109.1
C8—C3—C2	109.3 (2)	C7—C10—H10A	109.1
C4—C3—H3A	109.2	O1—C11—C12	121.17 (16)
C8—C3—H3A	109.2	O1—C11—C1	122.46 (16)
C2—C3—H3A	109.2	C12—C11—C1	116.20 (15)
C3—C4—C5	109.4 (2)	N1—C12—C11	113.06 (14)
C3—C4—H4A	109.8	N1—C12—H12A	109.0
C5—C4—H4A	109.8	C11—C12—H12A	109.0
C3—C4—H4B	109.8	N1—C12—H12B	109.0
C5—C4—H4B	109.8	C11—C12—H12B	109.0
H4A—C4—H4B	108.2	H12A—C12—H12B	107.8
C4—C5—C9	109.9 (2)	C14—C13—N1	122.10 (16)
C4—C5—C6	109.24 (19)	C14—C13—H13A	119.0
C9—C5—C6	109.26 (19)	N1—C13—H13A	119.0
C4—C5—H5A	109.5	C13—C14—C15	119.97 (17)
C9—C5—H5A	109.5	C13—C14—H14A	120.0
C6—C5—H5A	109.5	C15—C14—H14A	120.0
C5—C6—C1	109.47 (17)	N2—C15—C16	122.28 (17)
C5—C6—H6A	109.8	N2—C15—C14	121.01 (17)
C1—C6—H6A	109.8	C16—C15—C14	116.71 (16)
C5—C6—H6B	109.8	C17—C16—C15	120.57 (16)
C1—C6—H6B	109.8	C17—C16—H16A	119.7
H6A—C6—H6B	108.2	C15—C16—H16A	119.7
C1—C7—C10	109.44 (19)	N1—C17—C16	121.23 (16)
C1—C7—H7A	109.8	N1—C17—H17A	119.4
C10—C7—H7A	109.8	C16—C17—H17A	119.4
			
C11—C1—C2—C3	177.81 (19)	C3—C8—C10—C7	61.0 (3)
C7—C1—C2—C3	−59.4 (2)	C1—C7—C10—C9	60.2 (2)
C6—C1—C2—C3	59.5 (2)	C1—C7—C10—C8	−60.5 (3)
C1—C2—C3—C4	−60.9 (3)	C7—C1—C11—O1	40.2 (3)
C1—C2—C3—C8	60.3 (3)	C2—C1—C11—O1	162.52 (19)
C8—C3—C4—C5	−59.1 (3)	C6—C1—C11—O1	−78.1 (2)
C2—C3—C4—C5	61.3 (3)	C7—C1—C11—C12	−144.55 (17)
C3—C4—C5—C9	58.7 (2)	C2—C1—C11—C12	−22.2 (2)
C3—C4—C5—C6	−61.2 (3)	C6—C1—C11—C12	97.25 (18)
C4—C5—C6—C1	60.4 (2)	C17—N1—C12—C11	−122.92 (17)
C9—C5—C6—C1	−59.9 (2)	C13—N1—C12—C11	58.3 (2)
C11—C1—C6—C5	178.04 (16)	O1—C11—C12—N1	0.6 (3)
C7—C1—C6—C5	59.4 (2)	C1—C11—C12—N1	−174.80 (15)
C2—C1—C6—C5	−59.5 (2)	C17—N1—C13—C14	0.7 (3)
C11—C1—C7—C10	−175.80 (19)	C12—N1—C13—C14	179.53 (17)
C2—C1—C7—C10	59.3 (2)	N1—C13—C14—C15	−0.6 (3)
C6—C1—C7—C10	−59.2 (2)	C13—C14—C15—N2	179.56 (19)
C4—C3—C8—C10	59.7 (3)	C13—C14—C15—C16	0.1 (3)
C2—C3—C8—C10	−60.7 (3)	N2—C15—C16—C17	−179.07 (18)
C4—C5—C9—C10	−59.4 (3)	C14—C15—C16—C17	0.4 (3)
C6—C5—C9—C10	60.4 (3)	C13—N1—C17—C16	−0.2 (3)
C5—C9—C10—C8	60.2 (3)	C12—N1—C17—C16	−179.00 (16)
C5—C9—C10—C7	−60.7 (3)	C15—C16—C17—N1	−0.4 (3)
C3—C8—C10—C9	−59.9 (3)		

### Hydrogen-bond geometry (Å, º)

Cg1 is the centroid of the N1/C13–C17 ring.

**Table:**

*D*—H···*A*	*D*—H	H···*A*	*D*···*A*	*D*—H···*A*
N2—H1*N*2···Br1^i^	0.84 (2)	2.73 (2)	3.499 (2)	153 (2)
N2—H2*N*2···Br1^ii^	0.85 (2)	2.56 (2)	3.393 (2)	169 (2)
C12—H12*A*···Br1^iii^	0.97	2.72	3.664 (2)	166
C17—H17*A*···O1^iv^	0.93	2.59	3.434 (2)	150
C14—H14*A*···*Cg*1^i^	0.93	2.94	3.608 (2)	130

Symmetry codes: (i) −*x*, *y*−1/2, −*z*+1/2; (ii) −*x*, −*y*+1, −*z*+1; (iii) *x*, *y*−1, *z*; (iv) *x*, −*y*+1/2, *z*+1/2.
